# Microsatellite/SSR dataset: characterization of pear cultivars of the German Fruit Genebank

**DOI:** 10.1038/s41597-026-07010-y

**Published:** 2026-03-07

**Authors:** Lea Broschewitz, Brigitte Schramm, Henryk Flachowsky, Erik Schulte, Monika Höfer

**Affiliations:** 1 Julius Kühn Institute (JKI) - Federal Research Centre for Cultivated Plants, Institute for Breeding Research on Fruit Crops, Pillnitzer Platz 3a, 01326 Dresden-Pillnitz, Germany; 2 Federal Plant Variety Office, Testing station Wurzen, Torgauer Straße 100, 04808 Wurzen, Germany

**Keywords:** Plant genetics, Plant genetics, Genetic markers, Conservation biology, Plant molecular biology

## Abstract

The German Fruit Genebank (GFG) is a decentralized network dedicated to the conservation and utilization of traditional cultivars of different fruit species in Germany. Within this framework, the German Pear Network was established to document and preserve pear cultivars, with a focus on those of German origin or historical significance. To assess genetic diversity and authenticity, two projects were initiated under the Federal Ministry of Food and Agriculture, involving molecular and pomological characterization of pear cultivars across eight partner locations. After extensive data validation, 1,945 samples across 421 molecular groups were used to compile representative genetic profiles per cultivar, ensuring accurate documentation of pear genetic resources. Additionally, ploidy levels were determined via flow cytometry, and international alignment of genetic data was conducted to assign Pyrus UNiQue genotype codes (PUNQ). The resulting dataset enhances the transparency and accessibility of genetic information for research, breeding, and conservation efforts.

## Background & Summary

The decentralised gene bank network German Fruit Genebank (GFG) manages fruit genetic resources in Germany. Its aim is the effective and long-term conservation and use of traditional cultivars of different fruit species for research, breeding and other purposes (e.g. processing, extensive fruit cultivation, house- and home gardening). It also provides information on pomological and horticultural traits. The focus is on cultivars of native fruit species, i.e. pear, with priority given to German cultivars and selections, cultivars with socio-cultural, local or historical ties to Germany and cultivars with important pomological traits. One network per fruit species will be established, i.e. the German Pear Network. In accordance with the objectives of the GFG, an inventory was carried out to assess the authenticity and diversity of pear cultivars, resulting in the present dataset^1^ The Institute for Breeding Research on Fruit Crops, which is one of the 18 research institutes of the Julius Kühn Institute (JKI), the Federal Research Centre for Cultivated Plants in Germany, is coordinating the GFG.

The Federal Office for Agriculture and Food, acting on behalf of the Federal Ministry of Food and Agriculture, is responsible for the award of contracts for surveys, inventories and non-scientific studies in the field of biodiversity. In this context, two projects were set up to molecularly and pomologically characterize the pear cultivars in eight partner locations of the GFG (grant numbers: 2819BE003 and 2820BE003). The eight participating partner locations were: (1) Hermann Cordes Nursery KG, (2) Competence Centre of Fruit Production – Lake Constance, (3) State Education and Research Institute for Viticulture and Pomology Weinsberg, (4) Julius Kühn Institute (JKI) - Institute for Breeding Research on Fruit Crops, (5) State Institute for Agriculture and Horticulture Saxony-Anhalt, (6) Federal Plant Variety Office – Testing Station Wurzen (FPVO Wurzen), (7) Nonprofit Association: Teaching and Research Institution for Horti- and Arboriculture and (8) Triesdorf Agricultural Education Centre (See also Table 1). The Federal Ministry of Food and Agriculture commissioned the Schlaraffenburger Streuobstagentur (Schlaraffenburger gGmbH, Aschaffenburg, Germany; https://schlaraffenburger.de/) for the pomological characterization and Microsynth ecogenics GmbH (Balgach, Switzerland) for the molecular characterization. Within the GFG, the FPVO Wurzen manages the pear network and took responsibility for organizing and executing the project procedures. In line with their respective contracts, the Schlaraffenburger Streuobstagentur (Schlaraffenburger gGmbH, Aschaffenburg, Germany; https://schlaraffenburger.de/) and Microsynth ecogenics GmbH (Balgach, Switzerland) detailed their procedures and findings in respective project reports^2,3^. The information of these reports was translated and compiled in this data descriptor.

**Table 1:** Geographic locations of the pear collection of eight partners of the German Fruit Genebank’s Pear Network.

Institution	Location in Germany	Latitude	Longitude
Hermann Cordes Nursery KG	Holm	53.6282705	9.73713268
Competence Centre of Fruit Production – Lake Constance	Ravensburg	47.766853	9.557799
State Education and Research Institute for Viticulture and Pomology Weinsberg	Weinsberg	49.151023565199885	9.289324807052983
Julius Kühn Institute (JKI) - Institute for Breeding Research on Fruit Crops	Dresden-Pillnitz	51.0012786	13.8832785
State Institute for Agriculture and Horticulture Saxony-Anhalt	Quedlinburg	51.8137	11.1997
Federal Plant Variety Office – Testing Station Wurzen	Wurzen	51.372889	12.763087
Nonprofit Association: Teaching and Research Institution for Horti- and Arboriculture	Müncheberg	52.521326704589264	14.124764819505414
Triesdorf Agricultural Education Centre	Weidenbach	49.2061945	10.6596004

Throughout the project, 2,001 samples were molecularly analysed and 1,997 pomological assessments were made. The molecular data was initially analysed by Microsynth ecogenics GmbH (Balgach, Switzerland) and clustered into molecular groups based on similarity of the individual samples. 426 molecular groups were identified and each molecular group is meant to align with a cultivar. With the integration of the pomological data, some samples were omitted due to lack of quality or unclear alignment between molecular and pomological information. After extensive data revision, 1,945 samples in 421 molecular groups had adequate molecular as well as pomological data present. These were subsequently used for the compilation of the representative genetic profile per cultivar. These samples correspond to 1,917 individual trees, taking replicates and reference DNA samples into account. Between one to three trees are assigned to 979 unique accession numbers. The representative genetic profile is compiled from the individual sample data per molecular group based on the majority rule per marker variant. This means that the representative genetic profile can be pieced together from several samples and becomes a synthetic fingerprint that might not be identified as an individual sample anymore but is still the best representation for that molecular group and cultivar. 387 out of 421 (ca. 92%) cultivar genotypes can be traced back to a measured accession. Reciprocally, for 8% an actual synthetic fingerprint is the representative genotype. In this context and for more transparency, the published dataset also contains information on the frequency of the representative allele variant in relation to the samples of the respective molecular group. Based on accessions of the JKI’s Institute for Breeding Research on Fruit Crops, ploidy levels were previously determined by flow cytometry and incorporated into the dataset (unpublished, JKI-ZO, Dresden-Pillnitz). Lastly, INRAE (National Research Institute for Agriculture, Food and the Environment, Paris, France) aligned the final SSR dataset with international data to provide Pyrus UNiQue genotype codes (PUNQ)^4^.

## Methods

### Data collection

The data collection was separated into two steps: (I) pomological and (II) molecular characterization. Data collection for both characterizations was based on tree location lists. The lists were previously provided by the GFG partners and contain information of their collections. The lists were distributed by the pear network manager FPVO Wurzen. The general location of the participating institutions across Germany is given in Table 1. Information of the tree location, cultivar name in the collection and accession number were used for aligning the collected data.

Pomological experts visited the field collections of the GFG partner locations during ripening season for several years to identify pear cultivars. More detailed information on the process is found in *Section 2. Pomological characterization*.

Prior to sample collection for molecular characterization (Section 3), FPVO Wurzen supplied the contractor Microsynth ecogenics GmbH (Balgach, Switzerland) with collection lists of each GFG partner location stating the individual trees to be tested. Leaf material was collected between June and August of 2021. During the sample collection, correct association between samples and trees was guaranteed by a barcode system. Each sample bag as well as the entry in the collection list was barcoded with a sticker. The sampled tree was respectively barcoded by a tag. Preferably two young leaves were samples, bagged, barcoded and stored in an isolated bag pack with dry ice. At the end of the day, samples were transferred into −80 °C storage. After the initial sample collection, a second round of testing was performed. For this, the samples were collected, barcoded and mailed via post from the respective partner locations to Microsynth ecogenics GmbH (Balgach, Switzerland).

The total amount of samples with pomological and molecular data were delivered by respective project reports^2,3^. In Table 2, the number of data entries per GFG partner location and the total is displayed. Inconsistencies are found in i.e. the time frame of the project where trees died before completed pomological assessment. After merging the datasets based on the tree locations, 1,945 samples were feasible for further analysis. These 1,945 samples had complete molecular and pomological data (unpublished, JKI-ZO Dresden-Pillnitz). Excluding eight reference DNA samples and technical replicates, this dataset represents 1,918 trees and 979 accessions.

**Table 2:** Distribution of samples after data collection.

	# final samples	# trees	# accessions	# samples with molecular data	# samples with pomological data
**Hermann Cordes Nursery KG**	167	166	95	173	184
**Competence Centre of Fruit Production – Lake Constance**	293	293	147	293	293
**State Education and Research Institute for Viticulture and Pomology Weinsberg**	46	46	24	48	50
**Julius Kühn Institute - Institute for Breeding Research on Fruit Crops**	250	250	132	256	243
**State Institute for Agriculture and Horticulture Saxony-Anhalt**	168	168	56	168	168
**Federal Plant Variety Office – Testing Station Wurzen**	261	243	109	268	270
**Nonprofit Association: Teaching and Research Institution for Horti- and Arboriculture**	242	242	137	249	250
**Triesdorf Agricultural Education Centre**	510	510	271	538	540
**References Brogdale**	8	0	8	8	0
**Total**	**1945**	**1918**	**979**	**2001**	**1998**

### Pomological characterization

The pomological characterization was conducted by pomology experts and members of the Schlaraffenburger Streuobstagentur (Schlaraffenburger gGmbH, Aschaffenburg, Germany; https://schlaraffenburger.de/2. Said members were Jan Bade, Hans-Thomas Bosch, Steffen Kahl, Jens Meyer and Alexander Vorbeck. Next to their field experience, the pomologists used historic literature, preferably primary source literature, for cultivar identification. The pear accessions in the GFG collection are already named at each respective partner location usually based on the provided sourcing information. Therefore, in most cases, the pomologist could initially confirm or deny the given cultivar name. Additional support was provided by personal field collections of pomologists Jan Bade and Jens Meyer. Furthermore, during the project, a seed collection was created and per cultivar, characteristic fruit samples were photographically documented.

Pomological cultivar identification mainly relies on recognizing a combination of phenotypical traits and comparing these finding with literature descriptions and personal experiences. Important characteristics for pear cultivar identification are the seeds, shape of the fruit and especially the stalk and its base. Furthermore, the ground colour and russeting are cultivar specific traits. The last two traits, for example, are eminently variable between years and therefore experts need to be able to account for a degree of variation during the cultivar assessment. The flavour of the fruit is usually not a clear indicator towards a certain cultivar but gives insight on the utilization i.e. the acid/sugar ratio differs between dessert and cider pears.

Common goals and steps of the pomological characterization were:

- Cultivar assessment by several pomologists
- Detailed study of characteristic of fruit samples
- Correlation between fruit samples and personal and literature cultivar descriptions
- Comparison between seed samples and seed collection
- Discussion and confirmation of pomological characterization
- Documentation of pomological characterization results in line with the GFG trueness-to-type criteria (Section 5)

### Molecular characterization

The molecular characterization was performed by Microsynth ecogenics GmbH (Balgach, Switzerland)^3^. A protocol of 17 SSR markers as suggested by the European Cooperative Programme for Plant Genetic Resources (ECPGR) working group Malus/Pyrus was used^5^. The ECPGR also listed eight reference genotypes: ‘Abbe Fetel’, ‘Conferencce’, Doyenné du Comice’, Passe Crassane’, ‘Williams’, *Pyrus calleryana* ‘Chantecler’, *P. pyrifolia* ‘Hosui’ and *P. salicifolia* ‘Pendula’. The reference genotypes used in this project were provided by UK National Fruit Collections (Brogdale, United Kingdom).

#### DNA extraction

The isolation of genomic DNA was performed with the NuceloSpin Plant II kit in 96-well plates (MACHEREY-NAGEL GmbH & Co. KG, Düren, Germany). A leaf punch of the individual samples was cut, lysed and DNA was extracted according to the manufacturer’s protocol. An aliquot of the isolation product was diluted for further PCR analysis while the undiluted isolate was stored at −20 °C. Each 96-well plate contained an isolation control (no leaf material), a PCR control (no DNA template) and a positive control (known sample).

#### Genetic analysis

For this genetic analysis, 17 simple sequence repeat (SSR) or microsatellite markers were used: CH01d08, CH01d09, CH01f07a, CH02b10, CH03d12, CH03g07, CH04e03, CH05c06, EMPc11, EMPc117, GD147, GD96, CH_Vf1, CH04c07, CH05a02, GD142, NZ05g8. Marker combinations were established into four multiplex PCR assays by Microsynth ecogenics GmbH (Balgach, Switzerland). Each forward primer was tagged with a fluorophore at the 5’-end. The specific fluorophores are detected by fragment analysis in capillary electrophoresis. Fluorescein 6-FAM was caught by the blue detection channel. Rhodamine-fluorophores ATTO532, ATTO550 and ATTO565 were recognised by the green, yellow and red channels, respectively. Detailed information on primer sequences, marker-fluorophore-combinations and PCR multiplex assays are displayed in Table 3. All primers were synthesized by Microsynth AG (Balgach, Switzerland).

**Table 3:** Primer sequences of used simple sequence repeat (SSR) markers with information of used fluorophores and multiplex PCR assays.

Multiplex	SSR marker	Fluorophore	Forward primer 5′-3′-sequence	Reverse primer 5′-3′-sequence
**M1**	CH02b10	ATTO532	CAAGGAAATCATCAAAGATTCAAG	CAAGTGGCTTCGGATAGTTG
	CH05c06	ATTO550	ATTGGAACTCTCCGTATTGTGC	ATCAACAGTAGTGGTAGCCGGT
	EMPc117	ATTO565	GTTCTATCTACCAAGCCACGCT	CGTTTGTGTGTTTTACGTGTTG
	CH04c07	FAM	GGCCTTCCATGTCTCAGAAG	CCTCATGCCCTCCACTAACA
**M2**	EMPc11	ATTO532	GCGATTAAAGATCAATAAACCCATA	AAGCAGCTGGTTGGTGAAAT
	GD142	ATTO550	GGCACCCAAGCCCCTAA	GGAACCTACGACAGCAAAGTTACA
	CH01d09	ATTO565	GCCATCTGAACAGAATGTGC	CCCTTCATTCACATTTCCAG
	GD96	FAM	CGGCGGAAAGCAATCACCT	GCCAGCCCTCTATGGTTCCAGA
**M3**	CH01d08	ATTO532	CTCCGCCGCTATAACACTTC	TACTCTGGAGGGTATGTCAAAG
	CH03g07	ATTO550	AATAAGCATTCAAAGCAATCCG	TTTTTCCAAATCGAGTTTCGTT
	CH01f07a	ATTO565	CCCTACACAGTTTCTCAACCC	CGTTTTTGGAGCGTAGGAAC
	GD147	FAM	TCCCGCCATTTCTCTGC	GTTTAAACCGCTGCTGCTGAAC
**M4**	NZ05g8	ATTO532	CGGCCATCGATTATCTTACTCTT	GGATCAATGCACTGAAATAAACG
	CH05a02	ATTO550	GTTGCAAGAGTTGCATGTTAGC	TTTTGACCCCATAAAACCCAC
	CH03d12	ATTO565	GCCCAGAAGCAATAAGTAAACC	ATTGCTCCATGCATAAAGGG
	CH-Vf1	FAM	ATCACCACCAGCAGCAAAG	CATACAAATCAAAGCACAACCC
	CH04e03	ATTO532	TTGAAGATGTTTGGCTGTGC	TGCATGTCTGTCTCCTCCAT

For the PCR amplification, HotStarTaq DNA polymerase (0.05 U/µl; QIAGEN, Hilden, Germany), 15 mM MgCl_2_, 200 µM of each dNTP, 1 mg/ml bovine serum albumin (New England Biolabs Inc., Ipswich, MA, USA) and 1% PVP40 (Sigma-Aldrich, Merck KGaA, Darmstadt Germany) were used. The cycler program started with denaturation for 10 min at 95 °C followed by 40 cycles of 0.5 min at 95 °C, 1.5 min at 55 °C (multiplex PCR 1 and 3) and 45 °C (multiplex PCR 2 and 4) and 1 min at 72°. Final elongation ran for 30 min at 72 °C.

The dye size standard for the fragment analysis was GeneScanLIZ500 (Applied Biosystems, Thermo Fisher Scientific Inc., Waltham, MA, USA). The analysis ran on the 3730xl DNA Analyzer (Applied Biosystems, Thermo Fisher Scientific Inc., Waltham, MA, USA) with the following settings: 10 s injection time, 1.6 kV injection current, 2100 s run time, 15 kV run current, 50 cm capillary length, POP7 Polymer, Dye set G5 filter.

### Clustering of genetic profiles into gene groups

The evaluation of the chromatograms that resulted from the fragment analysis was performed in the GeneMarker software V2.6.4 (SoftGenetics LLC., State College, PA, USA) and each samples’ accuracy was checked. Based on the reference genotypes, an allele grid was determined for each marker to achieve robust allele detection throughout the study. Sample analysis with GeneMarker was only carried out when all quality controls were passed and dye size standard was clearly assigned.

The resulting genetic profiles were compared and grouped together. The pairwise distance between samples is derived from the Dice-Sørensen coefficient^6,7^ and was calculated based on Eq. 1.

1 $${pairwise}\,{distance}=1-\left(\frac{2\times \sum {common}\,{alleles}}{\sum {alleles}\,{sample}\,1+\sum {alleles}\,{sample}\,2}\right)$$

Small differences in allele fragment lengths (AFLs) are fairly common during the determination of allele calls and therefore deviation of ±1 bp was allowed in the calculation of pairwise distance. The cut-off for defining the molecular groups was set at 0.2 or 20%. So, samples that share 80–100% identity are grouped together. The cut-off was chosen based on the distribution of the pairwise distances across the whole dataset. The calculations for the pairwise distances and clustering analysis were performed in R software and with an internally developed Python script^8,9^. The molecular groups were named with the scheme of “Pyr_” as a precursor in relation to the *Pyrus* genus, followed by a four-digit running number, e.g. Pyr_0001.

### Trueness-to-type criteria

The trueness-to-type criterion was established by the GFG to indicate the certainty of cultivar identity. This criterion is compiled based on the combined pomological and molecular data, while the pomological assessment takes greater importance in this process. The criteria range from “0” to “5” and are shortly explained in Table 4. A more detailed explanation can be found in the data descriptor by Broschewitz, *et al*.^10^.

**Table 4:** Explanation for trueness-to-type criteria as used in the German Fruit Genebank (GFG).

trueness-to-type GFG criteria		Explanation
0	Deceased	tree deceased during project duration
1	true-to-type	pomologically and molecularly assessed
2	true-to-type (group/mutant)	pomologically and molecularly assessed
3	not determined	no references available, cultivar name unknown
4	not assessed	not assessed (pomologically)
5	true-to-type with reservations	pomologically assessed, not assessed molecularly
		molecularly assessed for at least 3 accessions and pomologically assessed for at least 2 accessions
		pomologically assessed with reservation, molecularly assessed

### A representative, genetic profile per cultivar

As mentioned previously the reviewed dataset contained 1,945 samples clustered into 421 molecular groups (unpublished, JKI Dresden-Pillnitz). Each molecular group should represent a cultivar. Here it has to be mentioned that sports (mutants) of a cultivar cannot be molecularly distinguished by the used SSR marker set, e.g. molecular group Pyr_0007 is aligned to cultivar ‘Gute Luise’ and its sport ‘Doppelte Gute Luise’. This is the case for a total of seven molecular groups or cultivars and their respective sports. In the dataset, the molecular group is represented by the originating cultivar name and the names of the respective sports as part of the GFG collection are listed in a separate column.

The number of samples per molecular group ranged from 1 to 35. The representative genetic profiles were compiled by the Python-script “synthetic_fingerprint_SSR_v2”^9,11^. This software groups the dataset per molecular group and picks the most common allele fragment length (AFL) per marker position. Per marker and position, missing values are removed based on the first position and failed SSR amplification to ensure that the least amount of missing data is passed onto to representative genotype. After this process, the most common AFL variant is transferred to the representative profile and it is pieced together with each position and marker. As there can be tie with no clear common value, a flag for manual review is implemented. The output-file is constructed in such a way that it has to be reviewed to clear out the ties by returning the flag ‘CHECK’ instead of the AFL value. Also, per genotype the marker where such an inconsistency occurs is listed. The trueness-to-type criterion is also compiled with this script but in a hierarchical order rather than by majority rule. Across samples, the trueness-to-type criterion was passed on in order of desirability based on GFG standards (“1 < 5 < 2 < 3 < 4 < 0”). The GFG wants to highlight the quality of its collection and if there is at least one true-to-type accession (“1”), the cultivar will be represented with that criterion.

Then, the manual review entailed two main cases were editing of the AFL variant combination was necessary:

1. Choosing the more heterogeneous AFL variant combination (described by Broschewitz, *et al*.^10^)
2. Choosing from two or more AFL variants: when two or more AFL variants were most common at a given marker or marker position, the difference between AFLs and overall consistency across the samples were taken into consideration. For example, marker CH02b10 has a perfect repeat type, so the amplified AFL variants should express an even difference between one another^12,13^. Furthermore, to keep the manual editing and reshuffling between sampled to a minimum, the representative profile was chosen from an existing sample if possible.

On the note of piecing together the representative genotype, it has to be highlighted that for some cultivars this genotype is truly synthetic. That means no sample across the molecular group completely aligns with the representative genotype but it still represents the overall group in the best way possible.

### Integration of quality check, ploidy level and Pyrus UNiQue genotype codes

After the manual review, two quality checks were performed of which the results are also published in the final dataset^1^. First, the dataset of representative genotypes with partly synthetic genotypes is blasted back against the individual samples. This way it is shown that 387 of 421 (ca. 92%) of representative genotypes also appear in at least one sample of the given molecular group. This alignment was done with Python-script “FuDiMa_wrapper”^14^. This software first converts the SSR marker data into binary data based on the AFL variants per marker. Then, each provided genetic profile is compared to each other and similarity is calculated based on Dice-Sørensen coefficient^6,7^. Molecular groups where no 100% match was found are consequently identified as synthetic. Respective information was provided in the dataset column “retraceable to accession”.

Secondly, the frequency of the chosen AFL variant was investigated with Python-script “synthetic_fingerprint_SSR_frequencies”^15^. Per marker position, the algorithm calculates the frequency of the represented AFL variant across samples of a given molecular group. The average score is also computed and exported. The resulting information can be found in the third tab of the data file. This information can indicate markers or genotypes with a high variability.

The JKI’s Institute for Breeding Research on Fruit Crops in Dresden-Pillnitz (Germany), previously analysed the ploidy levels of certain accessions of their pear collection by flow cytometry (unpublished, JKI-ZO, Dresden-Pillnitz). This information was transferred to the genotype level and incorporated into the final SSR dataset.

Lastly, the final SSR marker data set was aligned with international data by INRAE (National Research Institute for Agriculture, Food and the Environment, Paris, France) to provide Pyrus UNiQue genotype codes (PUNQ)^4^.

Overall, this process was automated as much as possible to be as objective and transparent as possible during the selection of the representative genotypes. The automation also saves time when processing a lot of samples. Several indicators for the quality of the dataset are provided.

## Data Records

This data descriptor is instrumental in enhancing the transparency and reproducibility of a dataset stored in the OpenAgrar repository (https://www.openagrar.de). The dataset can be identified by the digital object identifier http-doi (10.5073/20250814-135212-0)^1^. The dataset contains representative SSR genotypes for pear cultivars of the GFG. Additionally, information on trueness-to-type, international alignment using the Pyrus UNiQue genotype code (PUNQ) and ploidy level is presented. The data (file: “Pear_SSR_dataset_250630.xlsx”) is stored as.xlsx-file format. The file contains four table sheets. The first table sheet presents the necessary identifying metrics i.e. cultivar name, molecular group and trueness-to-type, and additional information on mutations, references, PUNQ and ploidy level as well as the SSR marker data. The second table sheet contains thorough explanations of the columns of the first sheet. The third table sheet contains the frequency information as described in Section 7. The fourth table sheet is explains detailed information on columns of the third sheet.

In the repository, an additional description file is deposited (“Description_pear_SSR_v1_250814.docx”). This describes additional information i.e. the differences to the predecessor version of the dataset^16^.

## Technical Validation

The pomological assessment was conducted by knowledgeable experts, with the requirement that several experts had to review all cultivars (Section 2). Additionally, the experts were asked to provide relevant literature references that supported their identification process, ensuring the use of credible and established sources. Furthermore, a detailed project report was required^2^.

The molecular analysis was performed by Microsynth ecogenics GmbH (Balgach, Switzerland) (Section 3). Experimentally, necessary controls were co-analysed and technical replicates were included. The genotypic profiles of the analysed reference genotypes are in alignment to published reference data^5,17^.

The raw dataset of combined pomological and molecular data was thoroughly reviewed by two independent people. The automated approach to compile a representative genotype per cultivar reduces processing time and enhances reproducibility. For unclear cases, a manual revision was responsibly performed. The check for truly synthetic genotypes showed that only 8% of representative genotypes cannot be reciprocally aligned back to individual samples. As an improvement to similar work by Broschewitz, *et al*.^10^, the dataset now includes detailed information on the frequency of selected AFL variants.

## Usage Notes

The published dataset provides curated and reliable SSR genotype information of pear cultivars preserved in the GFG collection. The dataset of representative genotypes can be used as a reference for future genotype alignments with the goal of cultivar identification. By providing the specific PUNQ number, alignment with international datasets is possible and referencing between databases and publications is simplified. SSR genotype data as such can be used for analysis on genetic variation and structure as well as parentage and pedigree analysis. On apple, a similar dataset was used to e.g. investigate the dynamics of genetic structure over time^18,19^.

Since the dataset was curated for use by the GFG, the preferred names of the pear cultivars are provided in German. Synonyms can be found individually on the GFG website (https://www.deutsche-genbank-obst.de/), which is regularly updated. Additionally, this dataset is biased toward German pear cultivars, as these are the primary focus of conservation efforts.

## Data Availability

The dataset described in this work is deposited in the OpenAgrar repository (https://www.openagrar.de). The dataset can be identified by the digital object identifier http-doi (10.5073/20250814-135212-0)^1^. There, the files “Pear_SSR_dataset_250630.xlsx” and “Description_pear_SSR_v1_250814.docx” are stored alongside additional meta data embedded in the repository website. A predecessor version of the discussed dataset can be found at 10.5073/20231220-114634-0^16^.

## Code availability

All used Python-scripts are published at zenodo.org^11,14,15^. Programming was done with Python V 3.10.14. The Python-script is run via the command line.
